# Technology-enhanced interprofessional simulation in higher education healthcare training

**DOI:** 10.4102/hsag.v31i0.3506

**Published:** 2026-10-08

**Authors:** Richard M. Rasesemola, Manoko P. Molabe

**Affiliations:** 1 Department of Nursing, Faculty of Health Sciences, University of Johannesburg, Johannesburg, South Africa; 2 Department of Emergency Medical Care, Faculty of Health Sciences, University of Johannesburg, Johannesburg, South Africa

**Keywords:** healthcare technology, interprofessional education, simulation training, virtual reality, Interprofessional simulation

## Abstract

**Background:**

Interprofessional education (IPE) prepares students from different health professions to work together in complex healthcare systems worldwide. Although widely valued, integrating IPE into discipline-bound health sciences curricula remains difficult, with barriers limiting meaningful interprofessional learning experience. Simulation-based education (SBE) addresses some challenges by providing risk-free environments to practice collaborative clinical skills and decision-making. However, gaps remain in adopting simulation-based IPE in higher education institutions.

**Aim:**

This study explored the experiences of management and lecturers adopting technology-enhanced interprofessional simulation (TEIPS) within the Faculty of Health Sciences (FHS).

**Setting:**

This study was conducted in the FHS of a higher education institution based in Johannesburg, South Africa.

**Methods:**

A qualitative, descriptive, exploratory and contextual design was used. Semi-structured interviews were conducted with purposively sampled FHS management and lecturers involved in SBE. Data were transcribed and analysed using the Matrix Building Framework approach.

**Results:**

TEIPS helps bridge limitations in traditional IPE by enabling personalised feedback, supporting measurable teamwork behaviours and creating environments where purposeful interdependence is essential for learning.

**Conclusion:**

TEIPS functions most effectively in a curriculum-embedded ecosystem supported by strong institutional leadership, sustained resources and structured faculty development initiatives.

**Contribution:**

This study addresses critical gaps in educator capacity building and the lack of practice-informed approaches to integrating digital innovations into IPE. It shows how purposeful interdependence, sustained faculty development and curriculum integration enable TEIPS as a measurable, sustainable and equity-enhancing IPE approach in diverse contexts in varied contexts.

## Introduction

High-quality interprofessional collaboration (IPC) in clinical practice is grounded in purposeful interprofessional education (IPE) during training (Neely 2025). As healthcare systems began to grow more complex, it became clear that no single profession could address the full spectrum of patient needs, prompting a shift towards collaborative practice models for delivering safe, efficient and patient-centred care (Bridges et al. 2011; Hassan et al. 2024). Interprofessional education, which is designed to prepare students from different health professions to learn with, from and about one another so they could work effectively together once in practice, emerged as the educational response to this shift. Interprofessional education is a critical strategy for breaking down traditional professional silos and fostering collaborative practice competencies, including communication, role clarification and shared decision-making (Patel et al. 2025; World Health Organization 2010). The demands of collaborative care created the need for IPE, and in turn, IPE has become the essential mechanism that equips and sustains effective teamwork in clinical practice (Alotaibi et al. 2024). Despite broad consensus on its value, the practical integration of IPE into traditionally rigid and discipline-specific health sciences curricula is a persistent challenge (Perri 2025). Logistical barriers such as scheduling conflicts, physical space limitations and the intensity of the required resources often constrain opportunities for meaningful, face-to-face IPE (Alshmaly et al. 2025). Consequently, students from nursing, medicine, pharmacy and other allied health professions graduate with limited experiential understanding of each other’s roles, potentially perpetuating fragmented healthcare care models in clinical practice (Castillo et al. 2025). Simulation-based education (SBE) has emerged as a powerful pedagogical answer to this challenge, providing a controlled, risk-free environment for learners to practice clinical and collaborative skills (Koukourikos et al. 2021). High-fidelity patient simulators and standardised patients have been extensively used to create realistic scenarios requiring interprofessional teamwork (Palaganas et al. 2020). The theoretical underpinnings of this approach are rooted in Experiential Learning Theory, which posits that knowledge is created through the transformation of experience, emphasising ‘learning by doing’ over passive reception (Kolb 1984), as well as Situated Learning Theory, which posits that learning is a social process occurring within authentic, real-world contexts rather than abstract, classroom-based instruction (Lave & Wenger 1991). The advancement of digital technologies has significantly broadened the scope of simulation practices, giving rise to technology-enhanced interprofessional simulation (TEIPS). Building on this expansion, technological tools such as virtual reality (VR), augmented reality (AR) and online collaborative platforms offer innovative solutions to longstanding barriers in IPE (Foronda 2021). By enabling immersive, scalable and reproducible learning environments accessible to geographically dispersed participants, these technologies contribute to the democratisation of high-quality IPE (Mustafa Kamal et al. 2019). Moreover, such modalities are closely aligned with the principles of virtual collaboration. Defined as technology-mediated interaction directed towards a shared goal, virtual collaboration is increasingly recognised as a core competency in contemporary healthcare delivery (Ying et al. 2025). A growing body of literature examines specific applications of these technologies in health sciences education. Virtual reality environments, for instance, allow learners to inhabit a shared virtual clinical space and interact with both the patient and each other’s avatars, practising communication and procedural tasks in tandem (Brachet et al. 2025; Chance 2025). Similarly, distributed simulation exercises using video conferencing and shared digital workspaces have been shown to improve team communication protocols and situation awareness among nascent healthcare teams (Myronuk 2025; Sung & Hsu 2025). Although existing studies frequently report positive outcomes such as enhanced learner satisfaction and improved self-efficacy, significant gaps remain in the evidence base. Notably, there is a scarcity of rigorous, longitudinal research that examines whether competencies developed through TEIPS are effectively transferred to real-world clinical practice or translate into improved patient outcomes (Zendejas et al. 2013). Furthermore, the perspectives and developmental needs of educators and facilitators in higher education who play a critical role in designing and managing these complex digital learning environments are insufficiently explored (Afonso et al. 2025). Compounding these gaps, theoretical frameworks that guide the integration of digital innovations into IPE are still in the process of refinement, limiting coherent and theory-informed implementation (Bell & Fredland 2020). Although existing literature identifies several important gaps in technology-enhanced TEIPS, this study specifically addresses two critical areas. There is limited empirical insight into educators’ capacity building needs for implementing TEIPS, as well as a lack of contextually grounded, practice-informed frameworks to guide its effective integration into IPE curricula in real-world higher education settings. Therefore, this study seeks to address the intersecting gaps of educator capacity development and practice-based integration of TEIPS, by exploring the experiences of management and lecturers involved in its implementation within a Faculty of Health Sciences (FHS).

### Aims and objectives

This study explored how management and lecturers within the FHS experience the adoption and implementation of TEIPS, with particular focus on faculty capability development and the practical integration of TEIPS into health sciences curricula to understand the institutional, pedagogical and operational factors that shape its implementation. The objectives of this study were to explore how management and lecturers navigated the integration of TEIPS into existing curricula to support the development of interprofessional collaborative competencies and to describe evidence-based insights that can inform future institutional strategies for strengthening IPE through technology-enhanced simulation.

## Research methods and design

### Study design

A qualitative, descriptive, exploratory and contextual design was used to capture rich, detailed accounts of how management and lecturers within the FHS engaged with the adoption and implementation of TEIPS.

### Setting

This study was conducted within the FHS at one university in Johannesburg, Gauteng Province of South Africa. This faculty is known for its innovative, problem-based teaching philosophy and its strong integration of practical clinical training through facilities such as the Health Training Centre and advanced clinical simulation laboratories and offering diverse accredited programmes through its 11 departments. This faculty is located within a major urban university in Johannesburg, Gauteng province of South Africa (University of Johannesburg 2025). This setting provides an ideal context for exploring IPE and the adoption of TEIPS because of their commitment to 4IR-driven educational innovation.

### Study population and sampling strategy

The population for this study comprised academic staff within the FHS at a selected university in Gauteng province, South Africa. A purposive sampling strategy was employed to recruit participants with direct, substantive involvement in SBE. The study targeted lecturers who had prior experience in the design, facilitation or implementation of IPE and TEIPS activities using this sampling strategy. The study recruited a sample of 12 key informants, comprising two senior members of the faculty management team and 10 lecturers engaged in the design, facilitation or oversight of simulation-based learning. These participants were drawn from five FHS Departments comprising Emergency Medical Care (EMC), Nursing, Podiatry, Chiropractic, and Medical Imaging and Radiation Sciences (MIRS). This sampling strategy ensured that participants possessed the requisite knowledge and experience to provide informed insights into the integration of TEIPS.

### Data collection

Participant recruitment was conducted by the primary researcher using institutionally affiliated email addresses. Invitations outlining the purpose and procedures of the study were distributed to FHS management responsible for teaching, learning and research, as well as to lecturers involved in SBE. The researcher is affiliated with the FHS; however, no direct supervisory or evaluative role was held over the participants. Participation was entirely voluntary, and interested individuals were required to respond independently to the invitation. To minimise potential bias and power imbalances, all interviews were conducted by an experienced external qualitative researcher, ensuring neutrality during data collection. To accommodate participants’ demanding schedules, individual online interviews were arranged at times convenient for them and carefully planned to avoid clashes with core teaching and academic obligations. All interviews were conducted online using either Microsoft Teams or Zoom. Interviews lasted 45 min – 60 min and were recorded with participants’ permission. A semi-structured interview guide was used, centred on the primary question: ‘What are your views and experiences regarding the integration of TEIPS within your curriculum?’ Additional probing questions were tailored to the study’s objectives to encourage rich, detailed narratives. Throughout the interviews, the facilitator employed established qualitative techniques to deepen engagement and enhance the quality of responses. These included active listening, strategic clarification, paraphrasing for accuracy, reflective questioning and periodic summarisation. This skilled facilitation supported sustained participant engagement and helped co-construct a nuanced understanding of the phenomena under investigation. Data collection continued until theoretical saturation was achieved with 12 participants.

### Data analysis

Data were analysed using the Matrix Building Framework proposed by Miles, Huberman and Saldaña (2019), which offers a systematic yet adaptable structure for qualitative thematic analysis. The framework was applied through iterative cycles of coding, categorisation and verification, comprising three interconnected phases. The first phase comprised initial coding, where verbatim transcripts of each interview were subjected to detailed, line-by-line coding. During this inductive stage, descriptive and conceptual codes were generated to capture emerging ideas, recurring patterns and meaningful insights embedded within participants’ accounts. This process ensured that the analysis remained grounded in the data and sensitive to the nuances of their experiences. This was followed by theme development, where the initial codes were then organised into conceptually related categories. Through constant comparison, abstraction, and refinement, these categories were synthesised into overarching themes and sub-themes that encapsulated the central dimensions of participant perspectives on the integration of TEIPS. The development of these themes was iterative, with codes and categories revisited to ensure conceptual clarity and representational accuracy. Lastly, verification and consensus, which involved an independent coder examining a sample of the transcripts together with the preliminary coding framework, were done to enhance analytical rigour. Divergences in interpretation, coding labels or thematic boundaries were discussed and resolved through consensus. Throughout all stages of analysis, reflexivity was intentionally maintained. The research team critically examined their professional roles and potential interpretive biases, using reflexive notes and ongoing dialogue to ensure that the final themes authentically reflected participants’ contributions rather than researcher assumptions.

### Ethical considerations

Ethical clearance to conduct this study was obtained from the University of Johannesburg, Faculty of Health Science Research Ethics Committee (No. REC-2455-2023). All participants received detailed information about the study and provided voluntary, informed consent before participating in interviews. Their autonomy was respected throughout, with the right to withdraw at any stage without penalty. Privacy and confidentiality were ensured by assigning codes to participants, securely storing data in password-protected files and requiring confidentiality agreements from external interviewers and the independent coder. The researcher further complied with the *Protection of Personal Information Act 4 of 2013* by safeguarding personal information and restricting data access to authorised research personnel only (South Africa, 2013).

## Results

The study participants comprised 12 key informants: Two members of the FHS management team and 10 lecturers actively involved in SBE. Participants’ details are presented in Table 1.

**TABLE 1:** Demographics of the participants.

Participant code	Department	Experience in higher education (years)	Experience in SBE (years)
P1	Nursing	9	7
P2	Medical Imaging and Radiation Sciences	4	3
P3	Emergency Medical Care	7	7
P4	Podiatry	4	3
P5	Faculty of Health Sciences Management	30	26
P6	Faculty of Health Sciences Management	23	10
P7	Medical Imaging and Radiation Sciences	10	5
P8	Nursing	5	5
P9	Complementary Medicine	4	4
P10	Chiropractic	3	3
P11	Nursing	6	5
P12	Emergency Medical Care	9	9

SBE, simulation-based education.

As reflected in Table 1, participants’ professional experience within SBE varied, ranging from 4 to 10 years. The FHS management participants possessed extensive institutional experience, ranging from 23 to 30 years within the university. The lecturers reflected a broad disciplinary spread, comprising three from Nursing, two from MIRS, two from EMC and one each from Podiatry, Chiropractic and Complementary Medicine. Collectively, participants’ SBE experience within the institution ranged from 3 to 26 years. Table 2 shows the themes and related subthemes that emanated during data analysis. Two major, interrelated themes, further elaborated by four distinct subthemes, which elucidate the pathways and prerequisites for developing collaborative practice through technology.

**TABLE 2:** Themes and subthemes.

Themes	Subthemes
Theme 1: Strategic institutional enablers for systemic integration	1.1 Leadership, vision and resource allocation 1.2 Faculty capability development as a core enabler of systemic interprofessional education integration
Theme 2: Implementing and evaluating technology-enhanced interprofessional education simulations	2.1 Intentional curriculum design for integrated, competency-driven interprofessional learning 2.2 Using simulation technology to make interprofessional collaboration observable, measurable and practically essential

### Theme 1: Strategic institutional enablers for systemic integration

This theme highlights that strategic leadership and the deliberate development of educator capability are important for the successful institutionalisation of TEIPS. Participants described how meaningful, system-wide integration requires leaders who articulate a clear vision, allocate sustained resources and translate high-level priorities into operational realities supported by adequate infrastructure, funding and technical systems. Alongside this structural leadership role, participants emphasised the need for faculty capability development, stating that educators require structured, pedagogically grounded training and protected time to build the specialised facilitation skills necessary for effective interprofessional learning.

#### Subtheme 1.1: Leadership, vision and resource allocation

This subtheme addresses the foundational role of institutional leadership in driving educational transformation emphasising how committed leaders must create a clear strategic vision, actively champion the shift in teaching methods and ensure the provision of sustained resources, including funding for essential infrastructure, ongoing technical support and necessary software. To support the above, the participants said:

‘For this [*TEIPS*] to move beyond a few passionate individuals, it must be in the faculty’s strategic plan. We need a dedicated budget line, not just leftover funds. Leadership must say: “This is a priority for how we train future healthcare workers,” then resource it properly.’ (P5, FHS Management, 26 years’ experience)

Another participant indicated that without dedicated resources and detailed logistical planning, the institutional vision remains an abstract concept disconnected from the daily challenges faced by the teaching staff. The participant critiqued the gap between strategic pronouncements and on-the-ground experience. The participant commented:

‘Management speaks about a “seamless virtual ecosystem,” but our operational reality is a patchwork of logins, incompatible platforms, and no shared calendar for booking simulation slots. The high-level vision must be connected to a practical, operational plan that the technical and administrative staff are resourced to implement.’ (P3, Lecturer, EMC)

Participants also highlighted the need for leaders to move beyond capital expenditure and commit to the recurrent funding and human resource development necessary for long-term success, framing sustainability as a continuous financial and operational commitment:

‘There’s a danger in leadership just buying the “shiny new thing” to tick a box. Real investment means funding the ongoing costs of software licences, maintenance, mentorship, training and the time for us to develop scenarios not just the initial hardware purchase. A one-off grant does not build a sustainable programme.’ (P8, Lecturer, Nursing)

#### Subtheme 1.2: Faculty capability development as a core enabler of systemic interprofessional education integration

Participants highlighted a critical institutional need for structured pedagogical training that extends beyond basic technical tutorials, emphasising that proficiency with simulation equipment alone does not prepare educators to facilitate meaningful interprofessional learning. Without strategic preparation, technology can shift from an enabler to a barrier, drawing educators’ attention towards system management rather than collaborative student engagement. Participants stressed that IPE facilitation must be recognised as an institutional competency, requiring a tiered and sequenced development programme that builds conceptual, practical and reflective expertise over time. They further highlighted that genuine capability development demands protected time, ongoing mentorship and multi-stage learning opportunities, as well as specialised skills in guiding team dynamics, debriefing decision-making processes and managing interprofessional interactions far more complex than merely operating simulation tools. One participant indicated the need for structured pedagogical training beyond just technical tutorials:

‘I was handed a hospital room with advanced manikin and laptops and told to facilitate. I knew nursing, but I didn’t know how to run a session where a student from different disciplines had to solve a problem together, and me operating advanced technology to give realistic manikin reaction, changing vital signs with each intervention given. We need formal training on the pedagogy of interprofessional facilitation, not just a onetime tutorial.’ (P11, Lecturer, Nursing)

Another participant reiterated that technology becomes a barrier without strategic preparation:

‘The technology works, but the learning dynamic falls flat because you’re focusing on managing the platform instead of guiding the team’s interaction. I left the session knowing the students missed a key collaborative learning opportunity, and that was on me. We need rehearsal spaces for us, the educators, first.’ (P8, Lecturer, MIRS)

Other participants indicated that IPE facilitation as an institutional competency requires tiered training and that faculty development requires protected time and multi-stage learning:

‘We cannot assume expertise in collaborative pedagogy. Our development programme must be mandatory and tiered starting with the “why” of IPE, then the “how” of facilitation in a tech environment, and finally, mentorship in actual simulation delivery. It’s a new core competency for our academic staff.’ (P6, FHS Management)

‘True faculty development isn’t a single workshop. It requires protected time, time to observe experienced facilitators, time to co-design scenarios with colleagues from other departments, and time to reflect on your own practice. Without the institution formally allocating that time, training will always be superficial.’ (P12, Lecturer, EMC)

Another participant highlighted that there is a need for specialised facilitation skills beyond operating simulation equipment:

‘I am perfectly capable of operating the simulation manikins. That is not the skill I lack. The skill I lack is knowing how to deconstruct a team’s decision-making process in a virtual debriefing or how to subtly intervene when a student from one discipline is dominating the conversation online. That is a different kind of expertise entirely.’ (P9, Lecturer, Complementary Medicine)

### Theme 2: Transformative shifts in educational practice and collaboration

This theme described a significant transformation in how IPE is conceptualised, designed and enacted, highlighting a movement away from ad-hoc simulation activities toward intentionally structured, collaborative and competency-driven educational practices. This shift involves re-examining the pedagogical purpose of technology, embedding IPE meaningfully within curricula and leveraging simulation tools to deepen, not merely deliver, interprofessional learning. This theme further emphasised that authentic collaboration must be intentionally cultivated across programmes through aligned curricula and structured progression, while technology-enhanced simulation environments create unique opportunities to make teamwork both practically necessary and empirically observable.

#### Subtheme 2.1: Intentional curriculum design for integrated, competency-driven interprofessional learning

Participants described a necessary shift towards purpose-driven, competency-aligned design of IPE, emphasising that technology should only be used when it meaningfully supports the intended interprofessional outcomes, the ‘why’ preceding the ‘how’. Participants further highlighted that interprofessional simulation (IPS) must evolve from a standalone activity into a coherent curricular thread, intentionally woven across programmes rather than delivered as isolated events. This requires formal integration of IPE into module descriptors, learning outcomes and assessment structures, ensuring that collaborative practice is recognised as core rather than optional. Furthermore, participants emphasised the importance of scaffolding IPS across year levels, building progressively from foundational communication skills to complex team-based clinical decision-making, enabling students to develop interprofessional competence through structured, longitudinal learning. Participants expressed the need for purpose-driven use of technology as follows:

‘There is a real pressure to just “use the new simulator,” but we must ask first: What specific interprofessional competency does this session address? Are we using VR because it is the best tool to teach shared decision-making, or simply because we, have it? The “why” must come before the “how.”’ (P4, Lecturer, Podiatry)

Another participant added that IPS should be added to a curriculum as a thread, not an isolated event:

‘Right now, IPS feels like an event, not a thread in the curriculum. For it to be meaningful, we need scheduled planning time with colleagues from Nursing, EMC, Podiatry to map our curricula together and find the natural touchpoints where a joint simulation would reinforce what each programme is teaching separately.’ (P8, (Lecturer, Nursing)

Another participant reflected that it is important to formalise IPE in module outcomes and assessments:

‘We cannot claim to develop collaborative practice if IPS is an optional add-on. It must be written into module descriptors with clear, assessable learning outcomes for example to demonstrate role clarification in a simulated patient handover so that it carries the same weight as any other core competency.’ (P5, FHS Management)

Another participant indicated that scaffolding of IPS should be done across year levels:

‘A one-off simulation in the final year is too little, too late. We need a scaffolded approach: Simpler interprofessional tasks in earlier years to build basic communication, leading to complex multimorbidity scenarios later. The technology allows for this progression, but only if we intentionally design it into the curriculum map across all years and departments.’ (P10, Lecturer, Chiropractic)

#### Subtheme 2.2: Using simulation technology to make interprofessional collaboration observable, measurable and practically essential

Technology-enhanced interprofessional simulation was described as a powerful mechanism for making collaboration both practically essential and empirically visible. Participants highlighted that simulation design, such as fragmented patient information, role-specific interfaces and programmable manikin responses, actively constructs scenarios in which no single profession can succeed independently. These environments create safe spaces for role exploration, reinforce professional boundaries and interdependencies and generate repeatable, observable data on team dynamics, enabling educators to systematically evaluate communication, leadership and collaborative decision-making in ways not possible in real-world clinical settings. One participant reflected that technology creates purposeful interdependence:

‘I design the scenarios, so the “patient’s” full history is fragmented. No one can work alone. IPS and simulation technologies enforces the rule that to succeed, they must pool their unique pieces of the puzzle. It makes collaboration a functional necessity, not just a nice idea.’ (P12, Lecturer, EMC)

Another reiterated that technology enables safe role exploration and boundary navigation:

‘The simulation platform creates a safe space for role exploration. It visually and procedurally reinforces the checks and balances, the distinct responsibilities, and the necessity of consulting outside one’s own expertise.’ (P9, Lecturer, Complementary Medicine)

Another participant indicated that technology produces evaluable, repeatable collaborative behaviour data:

‘With the manikin’s programmable responses, one can recreate the exact moment a patient’s condition deteriorates for ten different teams. Each team’s response who speaks first, who assumes leadership, how information is shared becomes the data. We can then compare and contrast these dynamics in debrief in a way that’s impossible with a one-off, real-world observation.’ (P7, Lecturer, MIRS)

### Measures of trustworthiness

To ensure the trustworthiness in this study, the researchers adhered to an established framework, which prioritises credibility, transferability, dependability and confirmability (Lincoln & Guba 1985). Credibility was strengthened through prolonged engagement, methodological triangulation, peer debriefing with experienced qualitative researchers and the maintenance of a reflexive journal to document analytical decisions. Transferability was supported by the purposive sampling of information-rich participants and the provision of thick, contextual descriptions of the research setting and participant profiles, allowing readers to evaluate the potential applicability of findings to other contexts. Dependability was achieved via a stepwise replication of the analytical process and an external inquiry audit, complemented by meticulous documentation of all research procedures, including a coding framework reviewed by an independent coder. Finally, confirmability was ensured through a comprehensive audit trail, the use of triangulation and ongoing reflexive journaling to bracket researcher presuppositions, thereby grounding the interpretations in the participants’ authentic experiences as evidenced by verbatim quotations.

## Discussion

This study highlights two mutually reinforcing conditions that determine whether TEIPS can be embedded successfully and sustainably in health sciences curricula. The first one relates to strategic institutional enablers for systemic integration, most notably leadership, vision and resource allocation, together with faculty capability development. The second one relates to transformative shifts in educational practice and collaboration, characterised by purpose-driven curriculum design and the use of simulation technology to render collaboration observable, measurable and practically essential. Together, these themes signal a maturation from ad-hoc, tool-driven activities to institutionally planned, competency-driven ecosystems that focus on longitudinal learning, cross-programme collaboration and rigorous evaluation of team performance. This trajectory aligns with contemporary guidance showing that simulation achieves its full value only when governance, infrastructure, pedagogy and assessment are intentionally coordinated around interprofessional competencies and patient-safe teamwork outcomes (Moabi & Mtshali 2022). Participants argued that institutionalisation of TEIPS depends on senior leaders articulating a clear vision, ring-fencing recurrent funding for resources and translating strategy into operational plans such as budget lines, coordinated scheduling and supported platforms. This stance is in line with strategic frameworks for SBE which elevate leadership and governance, preparation and planning, training and development and finance as crucial pillars of sustainable, faculty-wide simulation strategies for health sciences education (Alghanaim et al. 2025; Novaes 2025). Leadership must convert lecturers’ ambitions into executable operations such as shared calendars for simulation sessions booking, integrated and interoperable digital platforms and serviceable workflows, so that lecturers can experience a coherent ecosystem rather than a patchwork of tools. This operationalisation requirement is also echoed in the national faculty-development guidance, which urges infrastructure planning, quality systems and multi-professional support, with explicit organisational recognition and resourcing of simulation laboratories (Alghanaim et al. 2025; Health Education England 2021; Taylor 2025). The broader educational technology literature across higher-education institutions shows that leaders who establish the vision, allocate resources, build infrastructure and recognise and reward staff members who integrate technology in their teaching and learning activities are decisive in moving from isolated innovators to organisation-wide adoption (Johnson & Brown 2025; Olaniyan & Uzorka 2024). Participants in the present study also warned against capital-only solutions such as the purchase of ‘shiny new’ hardware without ongoing investment in software licences, maintenance, technician support, mentorship and educator time. This critique aligns with recent consensus work that places stability and sustainability at the centre of SBE strategy and embeds simulation in routine institutional budgeting and workforce development, not in one-off grants (Alghanaim et al. 2025). Simulation standards similarly emphasise planned evaluation, faculty development and human-factors expertise. These are elements that carry recurring costs and cannot be delivered by hardware investment alone (INACSL Standards Committee 2021). Participants emphasised the inadequacy of brief tool tutorials and the need for structured, tiered, pedagogical development that starts with the ‘why’ of IPE, advances to the ‘how’ of facilitation in technology-rich environments and culminates in mentored practice and reflective debriefing. This stance is well supported in the literature. A scoping review of IPS in emergency care highlights faculty development as crucial and calls for assessment tools specific to interprofessional competencies (Barton, Lackie & Miller 2023). Other sources describe IPS as situated at the intersection of SBE and IPE. It emphasises the importance of using theories, frameworks and competency models to inform the design, delivery and evaluation of IPS while highlighting faculty development as an essential foundation (INACSL Standards Committee 2021). Emerging evidence shows that simulation-based faculty development improves educators’ knowledge and perceptions of IPE (Hernlen et al. 2022). National guidance from England also reinforces the need for rigorous, supportive and sustainable educational environments, noting that employers must provide simulation faculty with protected time, mentorship and structures for shared practice (Health Education England 2021). Competency-based IPE guidance similarly calls for systematic, evidence-informed design and team-training approaches, drawing on team science research showing that structured training improves learning, performance and patient outcomes. This strengthens the argument that IPE facilitation is an institutional competency requiring protected time and staged development (Bogossian et al. 2023). Therefore, institutions of higher learning should adopt tiered faculty development covering conceptual grounding in IPE and team science, technical-pedagogical integration, mentored facilitation with feedback and communities of practice for scenario design and reflective debriefing (Health Education England 2021; INACSL Standards Committee 2021). In addition, these institutions should also allocate protected time and create rehearsal spaces to ensure technology enhances, rather than replaces, effective facilitation (Hernlen et al. 2022). Participants also advocated scaffolded progression across year levels from foundational interprofessional communication to advanced, multimorbidity teamwork supported by technology that enables developmental growth. This aligns with competency-based guidance emphasising sequenced learning pathways and transportable competencies across professions and stages (Danielson & Willgerodt 2018). Likewise, simulation standards frame simulation-based IPE as a purposeful, staged pedagogy in which objectives, team roles, fidelity and debriefing are tailored to developmental level, presupposing deliberate curriculum mapping rather than reliance on a single event (Batley-Heath 2025). Therefore, in practice, curriculum committees should map interprofessional competencies longitudinally by identifying which IPS experiences align with specific competencies, clarifying where these experiences are situated within modules and outlining how they will be assessed (Patel et al. 2025; Williams et al. 2025). To support this integration, shared calendars and centralised scenario banks can facilitate coordinated cross-programme planning and ensure consistent, equitable access to interprofessional learning opportunities. Participants further reflected that using simulation technology makes collaboration observable, measurable and essential. Technology can help engineer purposeful interdependence, enable psychologically safe role exploration and generate repeatable behavioural data. Fragmenting patient information and aligning role-specific interfaces makes collaboration a functional requirement, allowing teams to instantiate and evaluate constructs such as leadership, shared mental models and communication with specific tools rather than relying on clinical happenstance (Saporito & Georgis 2026). Simulation spaces that reinforce checks, balances and boundaries promote consultation beyond one’s expertise; structured practice and feedback improve team performance and correlate with better outcomes (Bogossian et al. 2023). According to Barton et al. (2023), repeatable, scenario-controlled conditions via programmable manikins and virtual environments support comparative analysis of turn-taking, leadership and information sharing, which are skills required for effective IPE. Simulation as an IPE strategy requires intentional design and evaluation to produce safer and more collaborative practitioners. Therefore, effective TEIPS requires a coordinated technical system, strategic leadership, recurrent resources, structured faculty capability and purposeful curriculum design, among others. Multilevel governance frameworks, alongside quality assurance and sustainability planning, are critical as a connective mechanism that would help transform discrete islands of different departments’ enthusiasm into an integrated institutional archipelago (Alghanaim et al. 2025). Cross-school evaluation infrastructure would enable evidence aggregation and development of shared calendars, integrated platforms and protected educators’ development time (Williams et al. 2025). Leadership and curricular threading are therefore equity strategies as much as efficiency measures, reducing optionality and unequal exposure (INACSL Standards Committee 2021; Patel et al. 2025).

### Strengths and limitations

Evidence from the literature documents many elements seen in this study. However, the strength of this study lies in its unique contribution, which relates to several novel integrative insights. Firstly, this study moves the conversation from capital expenditure to capability and continuity, detailing how recurrent financial resources, mentorship and protected faculty time, among others, underpin sustainable TEIPS. Secondly, this study reframes purposeful interdependence as a design principle, so that collaboration becomes functionally necessary and produces evaluable, comparable behavioural data. This extends standards and reviews by articulating the scenario mechanics needed to translate competencies into observable performance. Thirdly, this study identifies educator rehearsal and facilitation micro-skills as targets for faculty-development frameworks and educator assessment, complementing evidence that simulation-based development shifts faculty knowledge and perceptions. Fourthly, this study frames curricular threading as an equity mechanism, ensuring that all students access interprofessional learning at appropriate developmental stages, directly addressing logistical and sociological impediments identified in the IPE literature. Fifthly, it positions simulation suites as measurement ecosystems for team science, foreshadowing AI-supported, multimodal analytics to provide adaptive feedback while calling for ethical, consent-based data pipelines and faculty preparation in observational methods. The limitations of this study relate to its design and context. This is a qualitative study that adopted a descriptive and exploratory design, situated within the contextual boundaries of a single higher education institution in Gauteng, South Africa, with participation limited to management and lecturers. Although the use of online individual interviews facilitated practical data collection, this approach presented inherent constraints, potentially limiting the depth of interpersonal engagement and the capture of non-verbal communication that face-to-face methods afford. The study’s findings are delineated by specific limitations, including a circumscribed sample size and its single institution focus, which consequently restrict the transferability and generalisability of the results. To extend this inquiry, future research would benefit from longitudinal designs to assess the sustained application of interprofessional communication competencies, alongside the integration of perspectives from clinical educators and practising healthcare professionals to enable more comprehensive analytical triangulation.

### Implications and recommendations

For practice, institutions should develop shared data standards, common rubrics for IPE, secure repositories of scenarios and ethical data pipelines that enable longitudinal, cross-programme analysis. Executive teams should embed TEIPS within institutional strategy by securing recurrent funding. For education, governance structures should formally link TEIPS decision-making to curriculum committees, timetabling offices, IT services and quality assurance processes. Such integration would ensure that purposeful interdependence, curricular threading and facilitation-focused faculty development are operationalised consistently and equitably, allowing scenario design, learning outcomes and assessment systems to align with pedagogical intent. Future research could focus on AI-enabled analytics and the impact of tiered faculty development on learning and clinical skills transfer. This would help advance simulation as an authentic ecosystem for collaboration among health professions students.

## Conclusion

Technology-enhanced interprofessional simulation emerges from this study not as a set of devices or isolated activities but as a systemic, curriculum-anchored ecosystem that depends on aligned institutional, pedagogical and technological enablers. Effective implementation requires leadership to convert strategic vision into operational structures with sustained resources and cross-programme infrastructure, while the faculty need tiered, mentored development that prioritises facilitation over technology handling. Longitudinal curriculum design is essential, threading interprofessional competencies across modules with aligned assessment, supported by simulation technologies that intentionally engineer interdependence and make team behaviours observable and comparable. These findings extend existing frameworks by identifying concrete design strategies that address common failure points. If adopted, recommendations from the results generated from this article promise more equitable access to meaningful IPE, stronger educator identities grounded in team science and institutional capacity to evaluate and iterate collaborative practice education with rigour.
